# When Life Got in the Way: How Danish and Norwegian Immigrant Women in Sweden Reason about Cervical Screening and Why They Postpone Attendance

**DOI:** 10.1371/journal.pone.0107624

**Published:** 2015-07-09

**Authors:** Fatima Azerkan, Catarina Widmark, Pär Sparén, Elisabete Weiderpass, Per Tillgren, Elisabeth Faxelid

**Affiliations:** 1 Department of Medical Epidemiology and Biostatistics, Karolinska Institutet, Stockholm, Sweden; 2 Department of Quality and Patient Safety, Karolinska University Hospital, Stockholm, Sweden; 3 Department of Learning, Informatics, Management and Ethics, Medical Management Center, Karolinska Institutet, Stockholm, Sweden; 4 Department of Community Medicine, Faculty of Health Sciences, University of Tromso, The Arctic University of Norway, Tromsø, Norway; 5 Department of Research, Cancer Registry of Norway, Oslo, Norway; 6 Samfundet Folkhälsan, Helsinki, Finland; 7 School of Health, Care and Social Welfare, Mälardalen University, Västerås, Sweden; 8 Division of Social Medicine, Department of Public Health Sciences, Karolinska Institutet, Stockholm, Sweden; 9 Department of Public Health Sciences, Global Health (IHCAR) Karolinska Institutet, Stockholm, Sweden; UNSW Australia, AUSTRALIA

## Abstract

**Introduction:**

Danish and Norwegian immigrant women in Sweden have an increased risk of cervical cancer compared to Swedish-born women. In addition, Danish and Norwegian immigrant women follow the national recommendations for attendance at cervical screening to much lesser extent than Swedish-born women. The aim of this study was to explore how Danish and Norwegian immigrant women in Sweden reason about attending cervical screening, focusing on women’s perceptions as to why they and their compatriots do not attend.

**Methods:**

Eight focus group discussions (FGDs) were conducted with Danish and Norwegian immigrant women living in Stockholm. The women were between 26 and 66 years of age at the time of the FGDs, and were aged between <1 and 48 years old when they immigrated to Sweden. A FGD guide was used, which included questions related to cervical screening, and obstacles and motivators to attend cervical screening. The FGDs were tape recorded and transcribed, and the results analysed according to the principles of qualitative content analysis.

**Results:**

The main theme was “Women have a comprehensive rationale for postponing cervical screening, yet do not view themselves as non-attenders”. Investigation of women’s rationale for non-attendance after being invited to cervical screening revealed some complex reasons related to immigration itself, including competing needs, organisational and structural factors and differences in mentality, but also reasons stemming from other factors. Postponing attendance at cervical screening was the category that linked all these factors as the reasons to why women did not attend to cervical screening according to the recommendations of the authorities.

**Conclusions:**

The rationale used to postpone cervical screening, in combination with the fact that women do not consider themselves to be non-attenders, indicates that they have not actively taken a stance against cervical screening, and reveals an opportunity to motivate these women to attend.

## Introduction

Cervical cancer is the third most common cancer among women worldwide, with a striking variation in incidence by geographic area [1]. Cervical cancer is more common in low-income countries, where it is a leading cause of cancer-related death in women [1, 2]. This is mainly due to a lack of screening programmes [3], which have proven successful in the prevention of cervical cancer. The purpose of cervical screening is to detect and treat asymptomatic pre-invasive lesions at an early stage, and its introduction has led to a decline in cervical cancer incidence [4–7] and mortality [7, 8] both in Sweden and in the rest of Europe. According to the national cancer strategy in Sweden, the recommended overall coverage of cervical screening is 85% [9]. However, overall coverage in the country is currently below 80% [10].

Besides Finland, most female immigrants in Sweden are from Denmark or Norway and these women have a relative risk of cervical cancer that is 80% and 70% higher, respectively, than that of Swedish-born women [11]. Only 47% and 44% of eligible Danish and Norwegian immigrant women, respectively, follow the national recommendations for attendance at cervical screening, compared to 62% of Swedish-born women [12].

Sweden and Denmark have a long history of organised cervical screening, as in these countries organised population-based screening programmes started in the 1960s, whereas screening in Norway was introduced in 1994. For this reason the reduction in cervical cancer incidence and mortality has been slower in Norway compared to Sweden and Denmark [13].

The Nordic societies are known for their universal welfare system and are considered to be very equal societies [14]. However, despite the general similarities between the three Scandinavian countries (Sweden, Denmark and Norway) cervical screening attendance in Sweden differs between Danish and Norwegian immigrant women and Swedish-born women. Studies among immigrant women in other settings have shown that factors such as acculturation, specifically language proficiency [15, 16], lack of health insurance [17–20] and cultural beliefs and values [21, 22] are obstacles to accessing health care services, including cervical screening. However, given the similarities between the Scandinavian societies, such factors are supposedly less common among Danish and Norwegian immigrants than other immigrants in Sweden.

A better understanding of underlying factors is important, as immigrant women who do not attend the cervical screening programme have a five-fold excess risk of cervical cancer compared those who do attend [12]. The aim of this study was to explore how Danish and Norwegian immigrant women in Sweden reason about attending cervical screening, focusing on women’s perceptions as to why they and their compatriots do not attend.

## Methods

### Cervical screening in Scandinavian countries

According to the recommendations of the Swedish National Board of Health and Welfare, women 23 to 50 years of age are advised to undergo cervical screening by Papanicolaou (Pap) smear every 3 years and women aged 51 to 60 years every 5 years. In Sweden, the health authority in each county manages the cervical screening programme, and information on both organised and opportunistic screenings are collected by the programme. The invitation, registration and follow-up systems are computerised and linked to the National Population Register, which includes all legal residents in Sweden. Eligible women receive an invitation letter from the organised screening programme, which in general includes an appointment time at a pre-selected antenatal clinic, and general information about the purpose of cervical screening. Women who do not respond to the invitation are re-invited every year until a Pap smear has been registered [23].

In Norway, a recommendation letter is sent encouraging women aged 25 to 69 years to contact their regular physician or gynaecologist to have a Pap smear if none has been recorded for more than 3 years, but the letter does not give an appointment time. A reminder is sent if no Pap smear is recorded within 1 year of the date of the recommendation letter. The screening uptake in Norway is about 76% for the targeted age categories, which is close to the aim of 80% in Norway [24].

In Denmark, women are invited to have a smear taken by a general practitioner [25]. Women aged 23 to 50 years are recommended to have a Pap smear every 3 years, and older women every 5 years until the age of 65, as long as the two most recent smears were taken within 10 years and were negative. The coverage in Denmark among women aged 23–59 is estimated at 69% [26].

Sweden, Denmark and Norway have many things in common, such as history, language, and social structure. Health services are also public in these countries, and, to a large extent, financed through taxes. Primary health care systems are well established, and hospitals with advanced specialised treatment and preventive services for women are also available in all three countries.

In this study, screening attendance was defined as attendance according to the recommendations of the Swedish National Board of Health and Welfare (Pap smear every 3 years at ages 23 to 50 years and every 5 years at ages 51 to 60 years). Non-attendance to screening does not necessarily mean that women have never participated. Non-attendance should be interpreted as non-adherence to screening recommendations, which can also include women who postpone their participation to screening.

In order to get a deeper understanding of women’s reasoning regarding cervical screening, a qualitative approach with focus group discussions (FGDs) was used. This method is suitable to collect data about attitudes, experiences, and perceptions [27], and can also be used for sensitive questions [28].

### Sampling and study participants

The target population for the present study was immigrant women aged 23 to 70 years from Denmark and Norway living in the Stockholm area. Since we aimed to get a deeper understanding of women’s reasoning regarding cervical screening in general, there was no special effort made to recruit only non-attenders. We also knew from our previous studies that less than 50% of immigrant women adhere to the recommendations of the Swedish National Board of Health and Welfare for cervical screening.

An updated list of women aged 23 to 70 years residing in Stockholm County was obtained from the Swedish tax authorities. The Swedish tax authorities comprise information about immigrant status (whether women were born outside Sweden and had immigrated, or were Swedish-born) [29].

Sampling was then done in two steps. First, we randomly selected 440 Danish (of 1258 eligible) and 400 (of 2612 eligible) Norwegian immigrant women from the list using a computer sampling program. These numbers were chosen based on previous experience of low response rates when recruiting FGD participants. The selected women were contacted by a posted letter and asked to participate in the study; they could respond by email or phone. As expected, very few women responded (<5). Therefore in a second step all randomly-selected women who had not yet responded were contacted by phone and asked if they were interested in participating in the study. We were unable to retrieve a phone number for 72 Danish and 98 Norwegian immigrant women, so these women were sent a second letter reminding them of the study and asking them to send their contact information. None of these women responded. Fifty-three Danish women and 28 Norwegian women accepted to participate in the FGDs, of which 26 and 15 women, respectively, were not able to attend at the last minute due to illness, work or other social priorities. Most women who were contacted by phone either did not respond, despite repeated calls, were not interested in participating in the study, expressed interest but were hindered by a variety of practical and logistical obstacles, or declined to participate in the FGDs but agreed to be interviewed individually (data from these women is not included in this report). In total, five FGDs with 27 Danish participants, and three FGDs with 13 Norwegian participants, were conducted (Table 1). Each focus group consisted of three to seven participants, who were assigned to an FGD by country of origin and age group (23 to 40 years and 41 to 70 years). The intention was to attain homogeneity in the group in order to facilitate an open atmosphere. Preliminary analysis was done continuously, in parallel with data collection. A minor change to the FGD guide was made in the initial phase of data collection.

**Table 1:** Characteristics of the participants.

	FGD^a^ (A)	FGD (B)	FGD (C)	FGD (D)	FGD (E)	FGD (F)	FGD (G)	FGD (H)	FGD (total)
Country of origin									
Denmark (N participants)	4	6	7	6	4				27
Norway (N participants)						6	4	3	13
Age (years, range)	29–37	42–66	27–39	30–40	44–66	26–37	52–66	41–53	26–66
The range of years since age at immigration (Number of women with missing information)	5–32	4–38	<1–25	19–35	16-48(1)	8-29(1)	21–48	22–39	<1-48(2)
Socioeconomic status ^b^									
High non-manual	4	1	4	2	3	3	3		20
Low non-manual		1	1	1				2	5
High manual		2		2		2		1	7
Low manual		1			1		1		3
Self-employed		1	1						2
Not in the labour force			1	1		1			3
Level of education ^c^									
≥13 years of schooling	4	2	5	3	3	2	3	1	23
10–12 years of schooling		3	2	3		3	1	2	14
≤9 years of schooling									
(Number of women with missing information of their level of education)		(1)			(1)	(1)			(3)
Children									
Yes	3	5	5	3	3	2	4	2	27
No	1	1	2	3	1	4		1	13
Attendance at cervical screening according to women (Yes/No)	4/0	6/0	6/1	6/0	4/0	6/0	4/0	3/0	39/1

^a^FGD: focus group discussion.

^b^Based on the socioeconomic index classification used in Census data.

^c^Based on the classification used inthe Swedish education system (SUN).

### Data collection

The research team, which consisted of midwives, public health experts, epidemiologists and a medical doctor, developed the FGD guide that was pilot tested on another group of immigrant women. The guide included questions related to perception of health, disease and prevention, cervical cancer, cervical screening and obstacles and motivators to attend cervical screening.

We enrolled two moderators, who were native speakers of Danish and Norwegian, respectively, to lead the discussions. The purpose of this was not to exclude women who were hesitant to participate due to language problems, and also to facilitate discussion by offering the opportunity for the participants to discuss in their own language if they so wished for clarity of thought. The Danish moderator worked as a research assistant and the Norwegian moderator was a midwife who worked in a clinical setting. The moderators were trained for 8 hours on two different occasions by FA, EF and CW in subjects related to cervical cancer and cervical screening attendance among immigrant women in Sweden, and how to handle different situations that could arise during FGDs. The FGD guide was also discussed with the moderators. Data was collected between April and May 2010 at Karolinska Institutet.

All participants completed a questionnaire with their background data before the FGDs started. The participants were also offered light refreshment prior to the FGD. They all understood Swedish, and were encouraged to speak Swedish during the discussion, although they were aware of the possibility to speak their native language if the need presented itself. When necessary, due to the more or less developed Swedish linguistic refractive of the immigrant women, clarification was requested by either the moderator or the assistant, either in Swedish, or in the native language. While it rarely occurred, if participants gave a response to a clarifying question in their native language, the moderator translated the response to Swedish and confirmed the translation with the participant. This was possible given the linguistic similarity between Nordic countries. Therefore, the transcripts used for this analysis contained Swedish-language responses only.

FGDs were tape recorded and began with a short presentation by the moderator, followed by a brief presentation of the study and the guiding principles of the discussion. The moderator led the discussion and made sure that every participant had a chance to voice their thoughts. An assistant (EF, CW, FA) took field notes, which included a description of the discussion environment, keywords of what the participants said and anything of value regarding the quality of the FGD with special attention paid to the interaction between participants. It was also the role of the assistant to ask follow-up questions on issues that needed clarification at any time during the FGD.

A summary based on the field notes and a debriefing session between the moderator and the assistant were also tape recorded immediately after each FGD. The FGDs lasted for 1 to 1½ hours, during which the women talked openly and freely. The moderator balanced the discussion so that all topics in the discussion guide were evoked. The FGD participants were offered the possibility to ask questions after the FGD, and if they were interested, they were provided with information on cervical cancer and screening, and told where to go if they had additional questions or needed to discuss any issues more in-depth. All participants were offered a gift as a token of appreciation; they could choose between two movie tickets or a 150 SEK gift certificate to a flower shop.

After finalizing the data analysis we used the personal identity number—a unique personal identifier assigned to every individual officially living in Sweden—to link the study participants with the National Quality Register of Cervical Cancer Prevention (NQRCP). The women’s attendance at cervical screening thus was analysed since time of immigration according to information from NQRCP (Table 2).

**Table 2:** Description of attendance at cervical screening of FGD-participants based on information from National Quality Register of Cervical Cancer Prevention (NQRCP)^a^.

	Denmark			Norway		
	No. of participants	Mean years of delay before Pap smear since time of immigration	Attendance at cervical screening according to NQRCP since time of immigration (Yes/No)	No. of participants	Mean years of delay before Pap smear since time of immigration	Attendance at cervical screening according to NQRCP since time of immigration (Yes/No)
Age group 23–40 years (No. of participants/ No. of total women)	17/27	2	16/1	6/13	<1	6/0
Age group 41–70 years (No. of participants/ No. of total women)	10/27	15	9/1	7/13	14	7/0

^a^Attendance at cervical screening is calculated according to the recommendations of the Swedish National Board of Health and Welfare. The time in mean years delay for all visits per women was calculated from the completion of the third year at ages 23 to 50 years and the fifth 5 year at ages 51 to 60 years. Non-attendance to screening does not necessarily mean that women have never participated. Non-attendance should be interpreted as non-adherence to Swedish screening recommendations.

### Data analysis

The recorded FGDs were transcribed verbatim. Data were analysed utilising principles of latent content analysis with an inductive approach [30, 31]. In this paper only data related to cervical screening, and obstacles and motivators to attend cervical screening, were included. The analysis of FGD transcripts was done by country of origin and by age group (23 to 40 years and 41 to 70 years). During the first step of analysis, the transcripts were read several times, making notes on the codes that arose from the content of the discussion, in order to become immersed in the data. Thereafter the identified codes were collapsed into broader categories, and finally one main theme was generated. The material was also read by CW, EF and PT and discussed several times with the first author (FA) during the analysis. Quotations from participants were chosen to support and illustrate the categories, and the generated theme. The quotes were translated from Swedish to English by a professional translator. Omitted phrases are indicated by “(…)”, and hesitation indicated by “…”, whereas square brackets "[] " indicate authors’ comments. The FGDs are labelled FGD A to FGD H (Table 1) and the participants in each FGD are numbered P1 to P7.

Examples of the analytical process are described and shown in Table 3.The process of analysis involved a back and forth movement between a detailed analysis of all parts of the text and an overall analysis of the whole data.

**Table 3:** Examples of the analytical process.

Meaning unit (participants quotes)	Condensed meaning unit	Code	Sub-Category	Category	Theme
[FGD D]“P2: I mean this is the way I think, I have so little…I should have done it [had the Pap smear] when I should have, and if I had been in Denmark then I would have had seven [Pap smears], I would have been there seven times. Now I attended once in seven years, sort of….	In Denmark had a pap smear once per year but after the move to Sweden had once in seven years.	Postponing cervical screening attendance-changed behaviour related to immigration.	Postponing cervical screening attendance	Postponing cervical screening attendanc	Women have a comprehensive rationale for postponing cervical screening, yet do not view themselves as non-attenders
So that…yes, I wasn’t proud of myself, no I am not.	Doesn’t feel proud not having had a Pap smear	Negative feelings not having had the Pap smear	Psychological factor	Previous experiences, psychological and individual factors	
P2: All the people were new and so was everything, for me it was like…went from being single to being married. Went from not having any children to taking care of three children. Went from studying to having a job. So everything was new all at once. So I was so completely exhausted when I went to bed and I didn’t dream for two years.	Feeling there were so many changes in life when moving to Sweden that you get a sense of exhaustion	Competing needs related to the move to Sweden	Competing needs related to immigration	Competing needs related to immigration	
And then you receive all this information in the letterbox, “welcome to this thing” and “welcome to that” and now you can become Swedish and everything in through the door. (…) Well it was a bit like that, that’s to say all this stuff that comes in through the door is impossible to relate to. There were so many other things you should….”	Hard to relate to all information that comes in through the door in the letterbox. There were so many other things you should. . .	Prioritisation of needs	Competing needs related to immigration	Competing needs related to immigration	
[FGD F] “P:5: I will not do it [have a Pap smear]-so I waited for the next invitation as I said and there was someone who said of course you should do it!”	Thought that no I will not do it [have a Pap smear]-so waited for the next invitation	Waited for the next invitation	Postponing cervical screening attendance	Postponing cervical screening attendance	Women have a comprehensive rationale for postponing cervical screening, yet do not view themselves as non-attenders
“P:5: I think it was my Mum who said it, you must go and do it, it is very important. Oh, it is? But does it hurt? (…)”	The Mum said you must go and do it [have a Pap smear], it is very important.	Reminded by a relative	Social support-motivation	Social support-motivation	
”P:4. I thought they explained very well why you should have a pap smear, so I went at once.”	Good explanation in the letter [why have a Pap smear]-went at once.	Good information in the invitation-motivates having a Pap smear.	Organisational and structural factors-motivation.	Organisational and structural factors- motivation.	
Moderator: What was it that made you think no? “P5: So the pain, does it hurt? I was worried about that.”	Worried to feel pain when having a Pap smear and therefore didn’t want to [have a Pap smear].	Afraid that having a Pap smear should hurt.	Psychological factor	Previous experiences, psychological and individual factors	Women have a comprehensive rationale for postponing cervical screening, yet do not view themselves as non-attenders
“P5: And I don’t know if there was anything in the letter to explain to me why, but anyhow I did not read it because I have a bit of problem reading and taking in information that I am not interested in. I don’t read it, it is difficult for me.”	Didn’t know if there was anything in the letter to explain why you should have a Pap smear, but did not read it anyhow. Have problem reading and taking in information that you are not interested in- it is difficult for you.	Tiresome read and understand written information.	Individual factor	Previous experiences, psychological and individual factors	
“P5: So no, I was just worried that it would hurt. But it wasn’t so bad when I had it done. I felt a little bit on. . .there were three spatulas they scraped with so now I feel that now yes. . .now I shall go.“	Worried to feel pain when having a Pap smear.It wasn’t so bad having a Pap smear. You felt a little bit so now you feel that you will have your Pap smear.	Afraid that having a Pap smear should hurt. Having had pap smear despite fear. Positive experience-motivated having Pap smear.	Psychological factor Previous experiences, psychological factor	Previous experiences, psychological and individual factors	

Omitted phrases are indicated by “(…)”, and hesitation indicated by “…”, whereas square brackets "[] " indicate authors’ comments. The FGDs are labelled [FGD A] to [FGD H] (Table 1) and the participants in each FGD are numbered P1 to P7.

### Ethics statement

The participants were informed about the aim of the study, that participation was voluntary, and were told where to turn to if they had questions. Verbal consent was obtained from all participants, and the FGDs were tape recorded only after acceptance by the participants. The Regional Ethics Committee in Stockholm approved the study. During the explanation of the guiding principles of the FGDs, the moderators also urged the women to respect the personal nature of the discussion, and to maintain confidentiality by keeping the information brought up during the FGDs within the group. The incentives used in this study are considered to be too small to have motivated women to participate [32], and were used more as tokens of appreciation. All summaries and presentations were conducted in such a way that no specific individual can be identified.

## Results

Danish women in the present study were between 27 and 66 years old. Their age at immigration to Sweden varied from less than 1 year to 48 years. The women from Norway were between 26 and 66 years old and between 8 and 48 years of age when they came to Sweden (Table 1). According to the questionnaire all participants had attended cervical screening at least once during their time in Sweden, except for one woman who had not attended screening neither in Sweden nor in the country of origin (Table 1). All participants spoke Swedish with more or less linguistic refractive.

During the analyses, one main theme: “Women have a comprehensive rationale for postponing cervical screening, yet do not view themselves as non-attenders” was generated from the categories that emerged from the data. This theme refers to how women who were invited to participate in cervical screening justified postponing it, from half a year up to a decade, according to their personal rationale. This rationale turned out to be quite complex, and included reasons related to immigration itself, including competing needs, organisational and structural factors, and differences in mentality, but also reasons stemming from other factors such as previous experiences, psychological and individual factors, childbearing-related factors, social support and social network, and risk perception (Table 4). Postponing attendance at cervical screening was a category that tied together the reasons to why they did not attend to cervical screening according to the recommendations of the Swedish National Board of Health and Welfare. Although these categories are presented separately in the results, women who participated in our study described these factors as being intertwined when discussing their reasoning for postponing cervical screening. The fact that the women postponed their attendance at screening and did not actively refrain screening built the main theme.

**Table 4:** Description of the codes and categories generating the main theme: “Women have a comprehensive rationale for postponing cervical screening, yet do not view themselves as non-attenders”.

Category	Codes (included in each category)
Postponing cervical screening attendance	• How the women referred to the delay in cervical screening attendance in different ways when reasoning about why they did not attend after receiving the invitation letter from the organised screening programme.
Competing needs related to immigration	• Prioritisation of other more urgent needs due to immigration. Behavioural change due to loss of routines from country of origin and many new routines in Sweden. Higher mobility pattern due to travelling or work abroad.
Organisational and structural factors	• Difficulties to navigate within the Swedish health care system. Perception of the invitation system for cervical screening as impersonal. Logistical challenges. Importance of relationship with the caregivers.
Differences in mentality	• Perception of large differences between the country of origin and Sweden. Reluctance of women to accept regular health controls and governmental involvement in private life. More anxious approach towards things you should or shouldn’t do in Sweden in comparison with a more easy going approach in the country of origin.
Previous experiences, psychological and individual factors	• Previous negative experiences in the health care system. More serious negative experiences in life, such as sexual abuse or rape. Negative emotions after being treated unprofessionally by health care personnel, fear of cancer or fear of disease and other negative feelings. Individual factors such as psychiatric disease, misconceptions about cervical cancer and cervical screening and reading disability.
Childbearing-related factors	• Reproductive years were referred to as an extra sensitive period in the women’s lives, accompanied by feelings of increased vulnerability. Women viewed the reproductive years as periods with more intense contact with the health care system, which conferred feelings of safety because of the regular checks-ups. Pap smear was not given as much importance among women who had passed their childbearing years when they moved to Sweden, compared to women who immigrated when they were in their childbearing years.
Social support and social network	• The impact of social support and network.
Risk perception	• The impact of age on risk perception. Women’s views of preventive efforts. Knowledge about cervical cancer and screening.

The information from NQRCP showed that both Danish and Norwegian women delayed their attendance at cervical screening in Sweden in accordance to the recommendations of the Swedish National Board of Health and Welfare (Table 2). Both Danish and Norwegian women in the age group 41–70 years delayed their attendance by 15 years and 14 years respectively. The delay was by 2 years and less than 1 year among Danish and Norwegian women in the age group 23–40 years. According to information from NQRCP all women had attended at least once in Sweden except for 2 Danish women.

### Postponing attendance at cervical screening

It was discussed in all age groups that women who were invited to participate in cervical screening justified postponing it, according to their personal rationale. As they described it, cervical screening attendance was given low priority at the time, and they therefore waited to attend, forgot about it, had the intention to attend later on, thought that they would attend after receiving the next invitation, or it was put aside, as one younger Danish woman explained:

[FGD D]

P1: Well I think that I received my first invitation letter at the age of 23 and I believe that it took me one or 2 years before I had my Pap smear. And I think it was just because I thought it would be unpleasant. To have the examination done. So I didn’t get another appointment and then I waited a while because…well, being quite young and I thought…that it wasn’t that important, so that’s why I waited a while to get there. I think I was about 25 when I had my first test [Pap smear].

### Competing needs related to immigration

Participants often postponed attendance at cervical screening due to other competing needs related to immigration. The prioritisation of these needs over cervical screening was comprehensively discussed among participants in the younger focus groups from both countries, but only superficially mentioned in the older age groups. The women described the competing needs related to immigration as overwhelming in the first years. One younger Danish woman described how her life so drastically changed when she moved from Denmark to Sweden that even everyday life became a challenge. She explained how difficult it was to deal with all the offers that came by mail, and that the invitation to cervical screening was just one of many other offers that she needed to deal with:

[FGD D]

“P2: All the people were new and so was everything, for me it was like…went from being single to being married. Went from not having any children to taking care of three children. Went from studying to having a job. So everything was new all at once. So I was so completely exhausted when I went to bed and I didn’t dream for two years. And then you receive all this information in the letterbox, “welcome to this thing” and “welcome to that” and now you can become Swedish and everything in through the door. (…) Well it was a bit like that, that’s to say all this stuff that comes in through the door is impossible to relate to. There were so many other things.”

Although younger Danish and Norwegian women were used to attending cervical screening in their country of origin, they described a behavioural change that they believed was related to their immigration to Sweden. In addition, some women elaborated on what happened when they immigrated, such as considerable changes in their routines, systems and processes, and loss of the routines that they were used to in their home country, as one of the younger Danish women explained:

[FGD D]

“Moderator: But you speak about changes in behaviour. In what way does the behaviour change when you move to another country?

P6: I can only say that it changes. I am not entirely sure why, there are new routines, there are new processes (…). Quite suddenly you forget, that what was a routine in the country you come from, suddenly doesn’t exist. There isn’t this pattern or routine.”

However, some of the younger Danish and Norwegian women cited a higher mobility pattern as a reason to postpone attendance at cervical screening after moving from their country of origin, either due to travelling, or to work abroad for shorter time periods. Other competing needs due to immigration, such as those related to a woman’s work situation, were also mentioned by women in all age groups. One older Norwegian immigrant woman stated how her intense life in Sweden being self-employed made health-related issues such as attending cervical screening less important, and how this delayed her attendance at cervical screening for an indefinite period:

[FGD H]

“P2:…there was a lot in life. There is a lot in life.

Moderator: But when it was like that, when there was a lot in life, it [the Pap smear] was given a low priority?

P2: Yes, it was. Health was given a low priority. It’s mostly in the last few years I have felt that I had time. In the last year I can say, that you can…that it’s easier, with work… It’s work and everyday life, so I haven’t experienced anything dramatic or anything. It’s just that it’s a lot.”

### Organisational and structural factors

In all the FGDs, women stated that initially they considered the Swedish health care system to be similar to that in their country of origin. However, when discussing the obstacles for not attending cervical screening in more detail, the women explained the differences they ultimately found between the health care systems, and how this influenced their attendance at cervical screening. The way the Swedish health care system is organised was cited as a problem, especially by women who were older at immigration, and who therefore might have had more difficulties navigating within a new health care system. Two older Norwegian women discussed how they perceived the differences between the health care systems in Norway and Sweden:

[FGD G]

“P2: And then when you come here it’s organised differently and then you like have to think in another way and if you are older then maybe it’s a bit more difficult to think in a different way or yes…find another way. (…)

P4: Yes, as you say, maybe a new system too, a new organisation.

P2: Yes, it’s a completely different organisation of health…health care. I think this is fairly clear to see here when you come.…”

In the same FGD, women discussed more in detail how differences in the structure of the Norwegian and the Swedish health care systems could have a negative impact on attendance at cervical screening. During the FGD, women explained that in Norway, the decision to have a Pap smear was taken in concert with the gynaecologist, and was a result of individualised care, in contrast to the organised invitation letter system in Sweden.

Immigrant women in all age groups discussed in detail how they perceived the cervical screening programme, both the invitation letter and the organisation of cervical screening, as impersonal. They made special reference to the lack of anonymity, when all women in a waiting room could be easily identified by the invitation letter in their hand. They had expectations of the Swedish health care system based on their earlier experiences in their country of origin, such as who the professional performing the Pap smear would be: a gynaecologist or a midwife, a male or a female, and a previously known or unknown person. Furthermore, it was said that the organised cervical screening programme in Sweden lacked the ability to deal with other health-related questions during the Pap smear appointment, which was in contrast to the women’s experiences in their country of origin. Some women said this had a negative impact on their attendance at cervical screening in Sweden. In addition, when women (FGD-participants) visited a doctor, be it their general practitioner or gynaecologist in their country of origin, they perceived that their doctor had a holistic view of their health status, and that they could discuss other health-related questions, not only those related to Pap smear, as some older Norwegian women described:

[FGD G]

“P3… I thought that I would go to a gynaecologist because in Norway a cell test [Pap smear] is taken by the gynaecologist, you don’t go to a midwife when you have it done, but you have it done by a gynaecologist. So I got there and everyone is just sitting there…(…). There were quite a few women sitting there and then…one went in and came out again after two minutes and the next one was on her way in before she came out. So it was like a conveyor belt. And then I felt that it was like…it was so impersonal. It was like that, it’s not me that…well, okay then. Let´s have it done. But maybe I had some questions and issues and enquiries (…)

P4: No, that isn’t why you go there.

P3: No, no.

P4: Just the test. (…)

P2: I think it’s fairly clear from this that the organisation and everything, plays a big role.”

Among older Danish women, organised cervical screening was specifically referred to as impersonal, and it was said that women were treated like cattle, as one Danish woman expressed it:

[FGD B]

“P2: I had a friend who was, she only went once, but when you came to this clinic you went into this cubicle with curtains in front. Then you had to sit there and wait. In, up in the stirrups, out again. She says she’s not going to do it again. It was like…it was as she said, it was like herding cattle.”

Participants in all age groups also discussed the logistical challenges of moving around in their new country, especially if other problems occurred such as non-professional treatment by health care personnel. Not being able to approximate the wait time was also mentioned as something that led to postponing attendance at cervical screening. This was discussed mainly in the younger age groups. One younger Danish woman said:

[FGD D]

“P2: Three hundred may come or I didn't know how many they called at the same time. What if there are 3 000 women on this particular day, so you could be sitting there with… So I have no [idea], I can’t understand the concept. So I went around with it, with this little slip of paper [in her bag for seven years].”

In contrast, there were also women in all age groups who discussed the benefit of being invited to cervical screening regularly, and how it gave them a feeling of security that they were being checked.

### Differences in mentality

Another reason why women did not attend cervical screening was explained by the differences regarding Danish/Norwegian and Swedish mentality. This was mentioned in all groups except older Norwegian women. The difference in Danish mentality was qualified as a more reluctant approach to regular health controls and governmental involvement in private life, as one older Danish woman described:

[FGD B]

“P6: Yes, but I think it also has a bit to do with the mentality of the Danes. That you don’t really have quite the same view on this, going for regular checks like the Swedes have. It’s rare in Denmark that you have this approach with planned, like every six months or every other year or every year that you go in for some check. It isn’t really in the same way in Denmark.”

One younger Norwegian woman defined the difference in mentality as an anxiety about things one should or shouldn’t do in Sweden, compared to a more easy-going approach in Norway. She considered this to be a reason why some women did not attend cervical screening:

[FGD F]

“P2: It’s a bit more nervous than in Norway, about everything and what you should do and what you shouldn’t do and what you should eat and what is harmful and yes, all of that. So it…I thought that perhaps it could be just, well ok, I haven’t heard about this in Norway so it must be just something the Swedes have invented [laughter].”

The difference in mentality was also described as having a more risky lifestyle, which was discussed only among younger Danish women:

[FGD A]

“P2: Perhaps a bit more, but if you say that we are Europe in the Nordic region [referring to a more easy going lifestyle], Danish. That maybe we smoke a bit more, drink a bit more, have a bit more…[laughter] fun. (…) No, but there is…and then after I moved here, there is a certain difference, mental difference no matter what they say in Denmark. That’s what you feel despite the fact that we are so close to each other as neighbouring countries…to attend to these screenings, no why should we do it. Or maybe you become a bit more rebellious; you don’t want to do it.

However, another participant in the same FGD rejected this argument and stated that not all Danish women, herself included, had a risky lifestyle:

[FGD A]

“P4: I don’t smoke, I very seldom drink, I think about my health, I attend screening. Okay, maybe I don’t fit into the image of a Danish woman who doesn’t attend screening. It’s just that…it’s just that everything is relative.”

Younger Danish women elaborated further as to how their expectations that Sweden and Denmark were similar were contradicted. These women perceived large differences when they moved to Sweden, and these differences led to overwhelming, energy-consuming experiences, as some younger Danish women commented:

[FGD D]

“P6: Yes, I was actually a bit surprised that after all it is a Nordic country, there can´t be that big of a difference, but there was a huge difference in everything. I also think that just because there is such a big cultural difference; it was also…it drained me of so much energy in the beginning.

P5: Beforehand you think that it would be just like living in Denmark, of course…

P2: Yeah right (laughter).”

### Previous experiences, psychological and individual factors

It was discussed in all age groups that prior negative experiences with the health care system and cervical screening programme in Sweden led to delayed contact with health care services even if there was a need. It also resulted in women seeking health care in their country of origin instead of in Sweden, as one younger Norwegian participant said:

[FGD F]

“P7: I have my family doctor here but should anything happen to me then I would call the health care centre in Norway [named the health care centre in Norway]…and speak with them, and then I would either have a phone discussion with…or…now we aren't at home very often, but if I were to take my coil [IUD] out then I’ll have an appointment when I am back home in Norway…”

This category also included more serious negative experiences in life, such as sexual abuse or rape. One woman’s experience of sexual assault led her to not seek any care. The woman stated, in contrast to the main theme, that she did not want to attend cervical screening at all. She had a determined view and explained in detail that she had been raped, and that was what kept her from attending cervical screening, as well as her view that concern about possible disease could have negative impact on her quality of life:

[FGD C]

”P4: I haven’t had any Pap smear taken, and I never will. That envelope goes straight in the bin. (…) But there is a reason why I refuse gynaecological examinations on the whole and that is a previous rape. (…) It is there, but then I also think why bother just in case you get ill? If you start to worry, you’ll reduce your quality of life and then you walk around with that worry for ages.”

The same woman argued:

[FGD C]

“P4: None of your business, yes. (…) Yes, that they should give a damn about me, more or less, you know, that it’s up to me whether I want…to do these checks. (…) I feel a bit like the state shouldn’t have control and it does feel a bit like this, we are controlled, we want to make sure that you stay healthy and not cost us money [laughter]. And I feel a little bit like it is up to me if I want to do different things.”

This category included psychological factors that women in all age groups talked about. Psychological factors were represented by both negative and positive emotional feelings that ranged from negative emotions after being treated unprofessionally by health care workers, to fear of cancer, or fear of disease without mentioning cancer explicitly. Other negative feelings discussed were those of defencelessness, exposure, or emptiness, as some older Norwegian women commented:

[FGD G]

“P3: Yes, it is unpleasant you can say…

P5: You feel very exposed. As you can’t defend yourself when you are lying…between your legs.

P2: With your naked backside [in full view].

P5: Literally. But then when you have had it done, it is…I always experience a feeling of emptiness, when they…when they release the neck of the womb [cervix], that yes, I have a feeling that it is empty, the first few minutes and then… That's the best way I can describe it. It disappears quite quickly then, I suppose it is when they have held the neck of the womb, that it is what makes…the feeling when it goes back again.

P3: No, I think it is unpleasant. I think it is very unpleasant, but then again…you know that soon it is done [laughter].”

Women also discussed individual factors that could influence attendance at cervical screening such as psychiatric disease, and misconceptions about cervical cancer and cervical screening. For example, one younger Danish woman wondered about the risk of getting cervical cancer just by having a Pap smear. A younger Norwegian woman stated that her fear of pain during Pap smear was an obstacle for her to attend cervical screening, but she also referred to her reading disability:

[FGD F]

“P5: So the pain, does it hurt? I was worried about that. And I don’t know if there was anything in the letter to explain to me why, but anyhow I did not read it because I have a bit of problem reading and taking in information that I am not interested in. I don’t read it, it is difficult for me.”

### Childbearing-related factors

This category focused on how childbearing might influence an immigrant woman’s decision to postpone attendance at cervical screening. This was discussed in all age groups. Women referred to time periods when they desired to become pregnant, were pregnant, gave birth, and began raising children as sensitive periods in their life, which could impact their attendance at cervical screening. A younger Danish woman reflected on the reasons why she did not attend:

[FGD D]

“P3: I don’t get it, even though I speak, write, read fluent Swedish and yet…and am intelligent and I just have to [be able to deal with] these type of things. But it was too much information. It was also a sensitive period for me, just finished a pregnancy, yes…a lot of things were going on, having a baby and all of that.”

In contrast, younger immigrant women also discussed how their desire to get pregnant motivated their attendance at cervical screening, as cervical cancer was perceived as a threat, especially if the woman had never given birth and was in her late thirties. Women in all age groups mentioned that pregnancy and childbirth were periods that coincided with more intense contact with the health care sector, which conferred a feeling of safety since they were checked very often. Some older Danish women mentioned that having children might act as a motivator for having regular contact with the health care sector. This was also described by older Norwegian women, but more in the context that cervical screening was motivated by the fact that a woman was of childbearing age at the time she moved to Sweden, whereas if she moved to Sweden at an older age and already had children, it was no longer an issue, and consequently cervical screening was not given as much importance.

It was also hypothesised among younger Danish women that women who have children might focus first on assisting their children in adapting to the new environment and as a result the woman’s own preventive health needs might be put last, as one younger Danish woman explained:

[FGD D]

“P6: Maybe it also has to do with when you come for example to Sweden and have three children, then the focus is on the children, getting them integrated into day care and school and friends and the system of dentists and…then you put yourself last.”

### Social support and social network

Social support and social network may influence a woman’s attendance at cervical screening. During FGDs our participants initially viewed their attendance at cervical screening as an independent decision. However, when the women’s experiences of cervical screening were discussed, social support and social network seemed to have an impact on their attendance. Some of the younger Norwegian women, for example, discussed reasons why they did not attend cervical screening when invited, and how their mothers influenced their attendance:

[FGD F]

“P5: I think it was my Mum who said it, you must go and do it, it is very important. Oh, it is? But does it hurt? (…) So no, I was just worried that it would hurt. But it wasn’t so bad when I had it done. I felt a little bit on…there were three spatulas they scraped with so now I feel that now yes…now I shall go.

P1: It was exactly the same for me. (…) So I…got an invitation and then I forgot all about it and then it was my Mum who said, but how does it work in Sweden, have you done this, have you been for a check? Do they check up on you? How…do you have a doctor? No, I don’t have a doctor, there’s nothing wrong with me. Well you have to do it, it’s important. So I just felt that I had to call and ask them to send one of these [invitations] to me, I suppose I have to go for this [laughter].”

Older Danish and Norwegian women discussed how the role of husbands/male partners might influence a woman’s attendance at cervical screening. Some women thought this might represent a negative influence if the husband/male partner didn’t think it was important for the woman to attend cervical screening. In contrast, younger Danish and Norwegian women conveyed how their male partners supported their attendance at gynaecological examination and cervical screening. For example, one younger Danish woman explained her husband’s firm encouragement for her to attend screening. He knew she had only had one Pap smear during her 7 years in Sweden, whereas she had had one Pap smear per year when she lived in Denmark. Younger Danish and Norwegian women also argued that social networks both at work and in private life could play an important role in a woman’s decision to attend to cervical screening. In particular, that the presence of a social network could support women who are trying to navigate within the Swedish health care system, while the lack of a social network could make it more challenging to attend to cervical screening, as one younger Danish woman said:

[FGD D]

“P3: But it’s also about networks. Because if you haven’t built up a network in Sweden then, if you move to Sweden, then you don’t get any suggestions about gynaecologists, dentists…all these things you have to look up, and then it isn´t done, because searching eniro.se [online telephone directory], well… [covers her eyes with her hands].”

### Risk perception

Risk perception was discussed in all age groups and was related to a combination of obstacles to cervical screening attendance that women perceived/experienced, and motivators that enabled their attendance. Risk perception comprised factors like the impact of age, women’s views about prevention, and knowledge about cervical cancer and screening. The association between age and risk of cervical cancer was multifaceted. On one hand, older age was said to be an increased risk for cervical cancer. On the other hand, some women associated older age with a low risk for cervical cancer if the woman had had normal Pap smear results in the past.

The association between older age and increased risk of cervical cancer was also said to be related to an increased risk of developing other diseases in general as one ages. Furthermore, the participants’ risk perception seemed to be connected to the health problems of people in their social circles, be they close or distant relatives, friends, or just people they had heard or read about. Indeed, women who knew others who had been affected by disease had two quite opposite reactions: either they wanted to attend cervical screening more often, or they postponed it, not wanting to know if they had cervical cancer. One older Danish woman described how she was convinced that in her family they didn’t die from cancer, but rather from other diseases:

[FGD B]

“P6: Something that has been a problem for me, is that once when I went it seemed like the nurse who had taken the test, she was more nervous about the results than I was. Because I know very well that my family doesn’t die from cancer, I’ve somehow taken that as a fact. My family dies from cardio-vascular diseases.”

It was apparent that most women considered themselves at low risk for developing cervical cancer, particularly younger Norwegian and Danish women. Therefore when they were invited to cervical screening they decided they could postpone their attendance. Younger Danish women discussed how they felt when they received the invitation letter:

[FGD D]

“P5: …well, it’s far too early. I’m still young [laughter]. Yes, I felt a bit like that’s something you get when you are old so…but I went there anyhow.

P1: Exactly. (…) You think well, this isn’t relevant to me, but you do it to be on the safe side.

P4: Yes, I dragged it out a bit, I think. Six months, maybe a year went by before I got round to it. But I knew, I saved the slip of paper and knew that I had to go.”

Women in all groups described that the risk was connected to sexual activity and number of sexual partners. One older Norwegian woman explained how she postponed attendance at cervical screening because she had had one normal Pap smear, and since she had had the same partner for many years after that, thought she had low risk of developing cervical cancer:

[FGD H]

“P2: I’m putting it off…I have it ahead of me…Yes, it has been on my mind, it has been on my mind (…). It might also be that maybe I have understood that it has something to do with sexuality, and I have had the same partner too, so that maybe I also didn’t feel that it was so serious for me to go there. If I hadn’t had a husband, but had different partners, then maybe I would have thought differently.”

Risk perception also seemed to be associated with limited knowledge about cervical cancer, screening, and prevention. Some women didn’t believe that it was necessary to have a Pap smear if they had no symptoms. So even if these women had knowledge about cervical cancer, it did not motivate them to attend cervical screening. One older Norwegian woman, working as a midwife, was generally positive, and advocated the idea of attending cervical screening. However, during the FGD she explained that as long as her menstruation cycle was normal and she did not have any symptoms, such as abnormal discharge, she postponed her own attendance:

[FGD G]

“P5: So that’s why I know I should go, then maybe you can find something in time, but so far everything is normal and then I say…but everything is surely normal and I don’t prioritise it this time. But then the next time I say, okay, I didn’t prioritise it last time so now I should do it.

P3: So you are going…?

P2: You keep notice of it…?

P5: Well…[laughter].”

## Discussion

Danish and Norwegian immigrant women who took part in this qualitative study revealed views that can be summarised into one main theme: “Women have a comprehensive rationale for postponing cervical screening, yet do not view themselves as non-attenders”. This view was apparent even when the delay had been up to a decade.

The main theme was generated from the categories that seemed to influence the delay in attendance at cervical screening: competing needs related to immigration, organisational and structural factors and differences in mentality, which were viewed by the women to be related to immigration, previous experiences, psychological and individual factors, childbearing-related factors, social support and social network and risk perception. However, for some women these factors were considered to enable cervical screening attendance. Although these categories are presented separately, the women described these factors as being intertwined when discussing their reasoning for postponing cervical screening, thereby revealing the underlying complexity of this issue. The women referred to the delay in attendance at cervical screening in different ways when reasoning about why they did not attend more promptly after receiving the invitation letter from the organised screening programme. Postponing cervical screening attendance was the category that linked all the reasons why women did not attend at cervical screening. The postponement was an interpretation of the data as the women did not seem to disregard cervical screening but only delayed their attendance.

Despite the many similarities between Denmark, Norway and Sweden with regard to the way of life, history, language and social structure, including the existence of an organised cervical screening programme [13, 14], immigration to Sweden per se seemed to influence the Danish and Norwegian immigrant women who participated in the FGDs not to attend to cervical screening according to the recommendations of the Swedish National Board of Health and Welfare. Consistent with previous research, competing needs have been shown to be major obstacles to cervical screening attendance among immigrant women [33]. However, the competing needs evoked by immigrant women in the United States were essential needs, such as food, shelter and clothing [33]. The Danish and Norwegian immigrant women in our study discussed specifically how their energy and focus were consumed by the change of environment, attempts to learn how different societal systems worked in the new country, and to their resettlement efforts, all of which are related to competing needs due to the immigration.

We found that organisational and structural factors negatively influenced attendance at cervical screening. The aspects brought up by the FGD-participants were differences between the health care system in the country of origin and that in Sweden, the perception of the cervical screening invitation system as impersonal, logistical challenges and the importance of having a relationship with caregivers. However, these factors have also been mentioned by Swedish-born women who have actively chosen not to attend organised cervical screening [34]. Therefore it could be argued that organisational and structural factors may have a negative influence on women’s attendance at cervical screening regardless of immigrant status.

The Danish and Norwegian immigrant women also discussed the reasons they delayed attendance at cervical screening related to previous experiences, psychological and individual factors and risk perception, which are also known obstacles among Swedish-born women [34,35]. A study among immigrant women in the United States also showed an under-utilisation of cervical screening due to previous negative experiences in the health care system [36]. The category of risk perception included the impact of age on risk perception, women’s views of preventive efforts, and knowledge about cervical cancer and screening. In our study, this category was found to be an important motivator to attend cervical screening, as has been shown in previous research [37, 38]. It has previously been reported that older Norwegian women in Norway perceive themselves to be at low risk of sexually transmitted cervical infections, and thus delay their attendance at screening [39], which is consistent with the reasoning of our Norwegian participants.

The participants in our study said that childbearing-related factors influenced women’s attendance at cervical screening, which is consistent with previous research [36]. However, in our study the reproductive years were not unanimously described as a period when they were more motivated to attend cervical screening. Indeed, some women referred to their reproductive years as an extra sensitive period in their lives, accompanied by feelings of increased vulnerability, which, on the contrary, seemed to delay attendance at cervical screening. The reproductive years were also viewed by the women as periods with more intense contact with the health care system, which conferred feelings of safety because of the regular checks-ups, thus causing some women to postpone their attendance at cervical screening. On the other hand, studies have shown that women who have passed their childbearing years attend cervical screening to a lesser extent [40, 41], as mentioned by older Norwegian and Danish women in our study. Consistent with previous research [39, 40], our results showed that the existence of social support and social networks are important aspects that can aid women in navigating within the Swedish health care system, whereas the lack of social support and network might make attendance more challenging.

The Danish and Norwegian women also discussed how differences in mentality influenced their attendance at cervical screening. In this category, the reasons why women delayed their attendance at cervical screening were related to their perception of large differences between the country of origin and Sweden, which led to overwhelming experiences that were energy-consuming. Other reasons discussed were the reluctance of women to accept regular health controls and governmental involvement in private life, and a more anxious approach towards things you should or should not do in Sweden in comparison with a more easy-going approach in the country of origin. To the best of our knowledge, no previous research has discussed how differences in mentality between the country of origin and the new host country influence attendance at cervical screening. The concept of acculturative stress, defined as the losses that occur when adjusting to, or integrating into a new system of beliefs, routines and social roles [42, 43], has been found to affect the lives of immigrants [42].

There are some limitations to this study. Firstly, the FGDs were held with a limited number of women in one urban area. The results can thus not be generalised to the larger Danish and Norwegian immigrant population in Sweden. However, obtaining a more profound understanding of how immigrant women reason about their attendance at cervical screening requires a qualitative approach. Secondly, we experienced difficulties in recruiting women, especially Norwegian women, and some women who expressed interest in participating in the FGDs were hindered by a variety of practical and logistical obstacles inherent to their life situations, which has also been reported in previous research [43]. Therefore we cannot be certain that we reached saturation (i.e. no more new information surfacing during the FGDs) for Norwegian women. Difficulties in recruiting hard-to-reach populations, such as minorities, pose challenges related to accessing and gaining the trust of potential participants [40]. In addition, although the participants in our study varied by socioeconomic status and also included women outside the labour force, the majority of them were of higher socioeconomic status no one had the lowest level of education. It is therefore possible that the results would have been more nuanced if participants with even more diverse backgrounds had participated. The low response rate in this study could also reflect that women who did not participate in the study may be those who do not attend at cervical screening. The self-selection among participants in the study could therefore have influenced our results. However, the analysis of the study participants postponement of their attendance at cervical screening according to the NQRCP was confirmed both among Danish and Norwegian women. Therefore, especially among the study participants in the age-group 41–70 years we do have representatives of women who do not adhere to the screening recommendations, since they postpone their participation to screening.

To facilitate an open atmosphere, we tried to attain homogeneity in group composition by forming groups according to country of origin and age. Moreover, we used native-speaking moderators to facilitate the participation of women who might have been hesitant due to language problems, but also to offer the participants the possibility to use their own language at times for clarity of thought. However, this possibility was seldom used. The discussions were open and lively and allowed different views to be expressed. This was also confirmed by reviewing the field notes taken after each FGD in the debriefing session. The multidisciplinary combination of the research team brought varied and practical expertise, as is recommended in migrant studies [44]. This team approach was also used in the data analysis aspect of the study in order to strengthen the trustworthiness of our categorisations and interpretations.

All study participants, except two women had attended cervical screening at least once between their immigration to Sweden and the date of the FGD. This might have contributed to their view of being attenders to cervical screening even though from a biomedical perspective and according to the recommendations of the Swedish National Board of Health and Welfare they were non-attenders.

It is interesting to note that although we approached immigrant women without knowing beforehand if they had attended cervical screening, the women discussed postponing their attendance, and what motivated this decision. Indeed, it can be sensitive to discuss non-attendance, especially since the norm in today’s Western societies is to take care of your health [45, 46], which for women includes having regular Pap smears [46]. Moreover, focus groups can be especially suitable as it is the groups’ and not one individual’s specific view that is presented, making women more comfortable to express their views [23], and increasing the likelihood that this study presents information both with regard to attenders and non-attenders within the immigrant groups studied. Furthermore, we believe the analyses of the women’s attendance at cervical screening according to NQRCP further strengthen the trustworthiness of the study.

The effects of moving to another country and the subsequent change in life situation in general are intertwined. To distinguish between these aspects was, however, not within the scope of the study, although some of the factors discussed among Danish and Norwegian women could be relevant for attendance at cervical screening regardless of immigrant status. In this study, we explored how Danish and Norwegian immigrant women in Sweden reason about attending cervical screening, focusing on women’s perceptions as to why they and their compatriots do not attend. Therefore, we cannot comment specifically on the participant’s views about health, disease, or knowledge about cervical cancer or prevention, which may also influence cervical screening attendance [47, 48]. There is a need for more research in these particular areas.

## Conclusions

The findings of the current study highlight various factors that could explain why Danish and Norwegian immigrant women postpone their attendance at cervical screening in Sweden. The main finding indicates that postponement of cervical screening attendance was based on the women’s rationale, which was partially explained by factors related to immigration, but was also influenced by other aspects, such as previous experiences, psychological and individual factors, childbearing-related factors, social support and social network, and risk perception. The women’s reasoning revealed no active stance against attending cervical screening, except in one case. This, combined with their unawareness, from a professional biomedical perspective, of being non-attenders, reveals an opportunity to motivate these women to attend. This study is a part of a growing accumulation of knowledge and expertise pertaining to immigrant women’s attendance at cervical screening and provides an insight and a greater understanding of how immigrant women perceive attendance at cervical screening in Sweden, and the challenges they face.

### Funding statement

Finally, the authors would like to thank their funders who made this study possible: the Health Care Sciences Postgraduate School, Karolinska Institutet (FA); the Swedish Council for Working Life and Social Research (CW), and the Swedish Research Council (EW, CW and FA).
